# Oxidative Stress and the KEAP1/NRF2 Axis in Saphenous Vein: Implications for Graft Patency

**DOI:** 10.3390/cells15060563

**Published:** 2026-03-20

**Authors:** Georgia R. Layton, Em Marston, Hannah L. Musa, Shameem Ladak, Alice Copperwheat, Akintoye Oluwanifemi, Ibrahim Antoun, Mustafa Zakkar

**Affiliations:** 1 Department of Cardiovascular Sciences, University of Leicester, Leicester LE3 9QP, UK; grl13@leicester.ac.uk (G.R.L.); em593@leicester.ac.uk (E.M.); ia277@leicester.ac.uk (I.A.); 2 Department of Cardiac Surgery, University Hospitals of Leicester NHS Trust, Leicester LE3 9QP, UK; 3 Leicester British Heart Foundation Centre of Research Excellence, Leicester LE3 9QP, UK; 4 Department of Cardiology, University Hospitals of Leicester NHS Trust, Leicester LE1 5WW, UK

**Keywords:** NRF2, KEAP1, vein graft disease, saphenous vein, oxidative stress, endothelial dysfunction, apoptosis, antioxidant signalling, nitric oxide, intimal hyperplasia

## Abstract

Vein graft disease remains a significant limitation to the long-term patency of venous conduits following coronary artery bypass grafting. Early oxidative stress, triggered by ischaemia–reperfusion injury and haemodynamic changes following the implantation of veins into the arterial circulation, disrupts endothelial integrity and initiates inflammation, apoptosis, and maladaptive remodelling. The KEAP1-NRF2 axis is a central regulator of cellular antioxidant responses; however, its role in the development of vein graft disease remains poorly defined. This narrative review aimed to summarise what is known about NRF2/KEAP1 signalling in modulating vein graft pathology. Methods: A systematic search of PubMed was conducted to identify original research studies examining the NRF2/KEAP1 pathway in human saphenous vein tissue in vivo or ex vivo. Narrative synthesis was performed due to limited evidential availability and study heterogeneity. Results: Only one study has directly evaluated NRF2 pathway activation directly in human saphenous vein tissue, and it demonstrated that Protandim (a herbal dietary supplement) treatment increased antioxidant enzyme activity and reduced oxidative stress markers, including superoxide and 4-hydroxynonenal, both known activators of MAPK-dependent smooth muscle proliferation. Adjacent studies in other cells and tissues reveal that NRF2 intersects with multiple pathways central to vein graft pathology: it suppresses NFκB-mediated inflammation, modulates eNOS-NO signalling, inhibits NADPH oxidase expression, regulates MAPK activation, and influences angiogenic responses. However, context-dependent activation of NRF2 under arterial cyclic stretch can paradoxically drive proliferation through p62-mediated KEAP1 sequestration and enhanced glutathione synthesis. Conclusions: The NRF2/KEAP1 pathway serves as a central integrator of oxidative stress responses that directly intersect with established mechanisms of intimal hyperplasia and pathological angiogenesis. Post-translational KEAP1 inhibition may offer a targeted intervention point to limit these processes. Critical gaps remain regarding our understanding of the role of NRF2 in human saphenous vein under physiological arterial conditions and sex-specific pathway regulation. Mechanistic studies in vein-specific models are essential for advancing our understanding and any potential therapeutic translation.

## 1. Introduction

Free radicals are highly reactive, unstable molecules containing unpaired electrons. They are generated by processes including mitochondrial respiration, nicotinamide adenine dinucleotide phosphate (NADPH) oxidase activity, and enzymatic oxidation. Reactive oxygen (ROS) and nitrogen species (RNS) serve essential physiological roles as primary antimicrobial effectors during the leucocyte oxidative burst, where their deliberate production and release represent a coordinated first-line defence against infection [1]. Beyond immune defence, ROS and RNS also function as intracellular signalling molecules regulating vascular tone, cell proliferation, and redox-sensitive transcription. It is only when their production exceeds the capacity of endogenous antioxidant systems, or when they are generated in the wrong cellular context, that they become drivers of pathology. Key pro-oxidant species include superoxide, hydroxyl radical, and nitric oxide (NO), all of which are true free radicals with unpaired electrons. Peroxynitrite, formed from the rapid reaction of superoxide and nitric oxide, is not itself a free radical but is a potent non-radical oxidant and nitrating species capable of causing extensive biomolecular damage [2]. When pro-oxidant production overwhelms antioxidant defence, the resulting oxidative stress disrupts cellular homeostasis and causes damage to DNA, lipids, and proteins [3]. Within the cardiovascular system, this imbalance contributes to disease progression, including impaired angiogenesis and endothelial dysfunction, which are central to vein graft disease (VGD) [4].

VGD refers to the progressive luminal narrowing of venous grafts following coronary artery bypass grafting (CABG) [5]. CABG is the surgical approach to bypass arterial obstruction in patients with coronary artery disease [5]. These autologous vein grafts develop flow-limiting stenosis due to intimal hyperplasia and vascular inflammation. The earliest insults to the vein arise through trauma from surgical extraction and manual distention to check for leaks from the graft [6]. This is followed by ischaemia as a result of the disruption of the vein’s vasa vasorum, as well as its prolonged submersion in preservation fluids before surgical implantation [7]. Finally, the implantation of the vein into a high-pressure arterial circulation subjects the vein wall to acute haemodynamic changes, including increased wall tension and a shift from low venous shear stress to higher arterial shear stress. Together, these insults induce endothelial dysfunction and activation, reduce nitric oxide (NO) production, and propagate oxidative stress and inflammation [8,9].

The cellular response to this oxidative burden is coordinated primarily through the kelch-like ECH-associated protein 1 (KEAP1)/nuclear factor erythroid 2-related factor 2 (NRF2) signalling axis. NRF2, a member of the cap ‘n’ collar (CNC) family of transcription factors, binds to antioxidant response elements (AREs) and induces numerous antioxidants and phase II detoxifying enzymes that contain ARE sequences in their promoters [10,11]. Under basal conditions, NRF2 is held in the cytoplasm by KEAP1, which targets it for ubiquitination and proteasomal degradation via cysteine residues sensitive to cellular redox status [11,12]. Oxidative stress, electrophiles, and thiol-reactive molecules disrupt KEAP1/NRF2 interactions, allowing for NRF2 nuclear translocation [13]. In endothelial cells, this pathway is also regulated by biomechanical forces, and its activation can suppress proinflammatory transcription [14,15]. The contribution of NRF2 to cardiovascular homeostasis is increasingly recognised, with its deregulation associated with endothelial dysfunction and vascular smooth muscle cell (VSMC) proliferation [16,17]. However, its specific role in the human saphenous vein and its intersection with established VGD mechanisms remains poorly defined. This narrative review critically evaluates current evidence linking NRF2/KEAP1 signalling to oxidative stress pathways known to drive intimal hyperplasia (IH) and associated pathological angiogenesis in vein grafts.

## 2. Methods

This review set out to identify all the literature evaluating the NRF2/KEAP1 axis in the context of the human saphenous vein. Inclusion criteria were all original research articles published in English in peer-reviewed journals which evaluated the human saphenous vein in vivo or ex vivo when exposed to any form of oxidative stressor. Studies were accepted if they evaluated any known form of oxidative stress exposure and/or any intervention known to mitigate oxidative stresses. Studies lacking any mechanistic insight into NRF2/KEAP1 signalling or its downstream targets were excluded, as were studies evaluating only in vitro cellular-level data.

Review articles, editorials, commentaries, or conference abstracts without original data were excluded. Although review articles were excluded from formal analysis, their reference lists were screened to identify any additional primary studies relevant to the objectives of this review. Electronic searches were conducted in PubMed using subject-specific keywords related to the NRF2/KEAP1 axis and the human saphenous vein. The comprehensive search strategy is reported in the Supplementary Materials. Author G.R.L. performed the database searches. Search results were imported into the Rayyan QCRI web app [18], and duplicates were identified and removed.

Three authors (G.R.L., E.M., and H.M.) performed a blinded, independent analysis of all papers identified in the initial search. Papers were initially screened by their titles and abstracts and then later, where necessary, their full texts to determine their relevance to the review. Conflicts were resolved through consensus among the two screening authors and the senior author, M.Z. Authors G.R.L., I.A., S.L. and M.Z validated the final selected papers for inclusion. A total of 267 studies were identified during the initial literature search, without duplication. All studies were screened against the inclusion and exclusion criteria, and only 1 was eligible. The absence of eligible studies directly examining NRF2/KEAP1 signalling in human saphenous vein tissue itself reflects a significant gap in the field. The remainder of this review therefore synthesises mechanistic evidence from indirect models, stratified by their proximity to the target tissue and haemodynamic context (summarised in Supplementary Table S1).

## 3. Established Pathways of IH and VGD

Several molecular mechanisms driving IH in vein grafts have been well characterised and involve multiple interconnected pathways.

### 3.1. ROS-MAPK Proliferation Axis

ROS, particularly superoxide and hydrogen peroxide, activate extracellular signal-regulated kinase (ERK) 1/2 and p38 mitogen-activated protein kinase (MAPK) pathways in vascular smooth muscle cells [19,20]. This activation drives VSMC proliferation and migration from the media into the intima, a central cellular process currently identified to be responsible for IH development. Lipid peroxidation products, particularly 4-hydroxynonenal (4-HNE), serve as secondary messengers that amplify MAPK signalling and promote sustained proliferative responses [5].

### 3.2. NFκB Inflammatory Cascade

Oxidative stress activates the transcription factor nuclear factor kappa-light-chain enhancer of activated B cells (NFκB), which upregulates inflammatory cytokines IL-1β interleukin-1β (IL-1β), tumour necrosis factor-alpha (TNF-α) and IL8, chemokines such as monocyte chemoattractant protein-1 (MCP-1) and adhesion molecules such as intercellular adhesion molecule 1 (ICAM-1) and vascular cell adhesion molecule 1 (VCAM-1) [21]. These inflammatory mediators recruit leucocytes to the vessel wall, perpetuating oxidative injury and promoting VSMC phenotype switching from contractile to synthetic, proliferative states.

### 3.3. eNOS-NO Dysregulation

NO is produced by endothelial cells and usually suppresses VSMC proliferation, inhibits leucocyte adhesion and prevents platelet aggregation [9]. Early endothelial injury occurring in vein grafts reduces the expression of endothelial nitric oxide synthase (eNOS) and therefore NO production and bioavailability. NO usually suppresses xanthine-derived ROS, so its loss impairs redox homeostasis. Thereby, paradoxically, loss of NO removes tonic suppression of NADPH oxidase, further amplifying ROS production and creating a vicious cycle of oxidative injury.

### 3.4. NADPH Oxidase–ROS Generation

NADPH oxidases, particularly NOX4, are primary sources of vascular ROS [22]. Their expression is upregulated in vein grafts by disturbed flow, inflammatory cytokines, and growth factors. The superoxide generated by NOX enzymes not only damages cellular components but also reacts with NO in a near-diffusion-limited reaction to form peroxynitrite, a potent non-radical oxidant. This reaction further reduces NO bioavailability and initiates a cascade of nitrosative and oxidative biomolecular damage [2].

### 3.5. Angiogenic Dysregulation

Whilst physiological angiogenesis supports vascular repair, pathological neovascularisation within the vein graft wall promotes IH by delivering inflammatory cells and growth factors to the intima [23]. It is the balance between pro-angiogenic signals such as vascular endothelial growth factor and hypoxia-inducible factor-1α (HIF-1α) expression and anti-angiogenic signals that determines whether vessel remodelling is adaptive or maladaptive. Unlike arteries, veins are physiologically adapted to a low-flow, low-shear environment and release significantly lower levels of shear-dependent vaso-protective factors, such as NO, prostacyclin, and tissue plasminogen activator, than their arterial counterparts [24]. Therefore, once implanted into the coronary circulation, the veins undergo a phenotypic transition termed arterialisation through proliferation and migration of smooth muscle cells into the intimal layer. Where this occurs due to disturbed non-laminar flow, it represents adaptive intimal thickening. However, sustained oxidative stress drives progression to pathological intimal thickening, which impairs coronary flow and causes clinically identifiable ischaemia.

## 4. The KEAP1-NRF2 Antioxidant Response System

**Figure 1:**
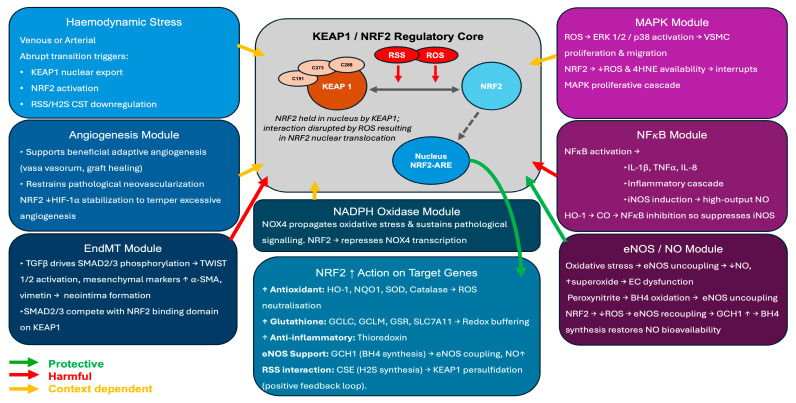
The core input mechanisms of the KEAP1/NRF2 axis in saphenous vein graft disease. The KEAP1/NRF2 regulatory core (centre) responds to both reactive oxygen species (ROS) and electrophiles produced under oxidative stress and reactive sulfur species (RSS), including hydrogen sulfide (H_2_S) and polysulfides. Both inputs modify cysteine residues on the KEAP1 dimer (C151, C273, C288), releasing NRF2 for nuclear translocation and antioxidant response element (ARE)-driven cytoprotective gene transcription. Protective outputs include antioxidant enzymes (HO-1, NQO1, SOD, catalase), glutathione synthesis enzymes (GCLC, GCLM, GSR, SLC7A11), anti-inflammatory thioredoxin, GCH1-mediated de novo BH_4_ synthesis, which supports eNOS coupling, and CSE-mediated H_2_S synthesis, creating a positive feedback loop. Surrounding modules illustrate the major pathological pathways operating in vein graft disease and the modulatory role of NRF2 in each. Haemodynamic stress arising from the abrupt venous-to-arterial transition activates NRF2 while simultaneously reducing H_2_S availability via CSE downregulation. NADPH oxidase (NOX4) propagates oxidative stress and sustains pathological signalling; NRF2 directly represses NOX4 transcription. NFκB activation drives inflammatory cytokine production, iNOS induction, and peroxynitrite formation, which uncouples eNOS in a self-amplifying loop; NRF2 suppresses NFκB via competition for CBP/p300 and HO-1-derived CO. In the eNOS/NO module, peroxynitrite oxidises BH_4_ to perpetuate eNOS uncoupling; NRF2 restores NO bioavailability through ROS reduction and GCH1-mediated BH_4_ synthesis. MAPK activation by ROS and 4-HNE drives VSMC proliferation and migration; NRF2 interrupts this cascade by reducing available ROS and 4-HNE. In the EndMT module, TGFβ-driven SMAD2/3 phosphorylation promotes mesenchymal transition and neointima formation; KEAP1 physically sequesters SMAD2/3, and NRF2 activation reduces SMAD2/3 phosphorylation, though direct investigation of this mechanism in LSV is lacking. NRF2 exerts a context-dependent role in angiogenesis, supporting adaptive vasa vasorum restoration while restraining pathological neovascularisation via HIF-1α suppression. Arrow colours denote signalling direction: green, protective or suppressive; red, pathological or activating; gold, context-dependent.

A key modulator of the vascular antioxidant response is the KEAP1-NRF2 signalling pathway. Under basal conditions, KEAP1 sequesters NRF2 in the cytoplasm, targeting it for proteasomal degradation. During oxidative stress, ROS modify cysteine residues on KEAP1 (particularly Cys151, Cys273, and Cys288), preventing NRF2 from being degraded. KEAP1 contains multiple redox-sensitive cysteine residues that act as sensors for distinct oxidant species. Hydrogen peroxide oxidises these residues to sulfenic acid (S-OH), which ntly condense with an adjacent thiol to form a disulfide bond (S-S) [25]. S-nitrosylation (S-NO), driven by nitric oxide and reactive nitrogen species, has also been demonstrated at the same sites. Electrophilic lipid peroxidation products, particularly 4-HNE, form covalent Michael adducts preferentially at C273 and C288. More recently, persulfidation (S-SSH), mediated by hydrogen sulfide (H_2_S) and polysulfides, has been identified as a further KEAP1 cysteine modification that promotes NRF2 stabilisation, a mechanism discussed in detail in the reactive sulfur species section of this review. Superoxide does not react efficiently with protein thiols directly but contributes to KEAP1 modification indirectly by dismutating to hydrogen peroxide. The specific modification at each cysteine sensor is not functionally equivalent: different modifications produce distinct conformational changes in the KEAP1 dimer and determine both the degree of NRF2 release and the subset of ARE-dependent target genes that are subsequently induced. This so-called ‘stabilised’ NRF2 translocates into the nucleus and binds to the antioxidant response element (ARE) in the promoter regions of key cytoprotective genes [26]. When cells encounter oxidative stressors, they activate intrinsic defence mechanisms to increase production of antioxidant enzymes such as haem-oxygenase 1 (HO-1) through ARE-dependent transcription [27] (Figure 1). Under basal conditions, NRF2 binds to ARE weakly. However, during oxidative stress, it dissociates from KEAP1, translocates to the nucleus and binds more strongly to the ARE, thereby initiating transcription of genes that neutralise reactive species and restore redox balance. These NRF2 target genes include phase II detoxifying enzymes: NAD(P)H quinone dehydrogenase 1 (NQO1), glutathione S-transferases, antioxidant enzymes (HO-1, superoxide dismutase, catalase) glutathione synthesis enzymes glutamate-cysteine ligase catalytic subunit (GCLC) and glutamate-cysteine ligase modifier subunit (GCLM) and those producing cytoprotective proteins such as ferritin and metallothionein. NRF2 activity is tightly controlled by protein–protein interactions beyond KEAP1. Competitors such as p62/SQSTM1 can sequester KEAP1, preventing the degradation of NRF2 [28]. Additionally, NRF2 is transcriptionally regulated by factors like NFκB, creating functional crosstalk between redox-sensitive and vascular inflammation signalling pathways.

### 4.1. Current Evidence for the Role of NRF2/KEAP1

To date, only one group have directly examined NRF2 pathway modulation in human saphenous vein tissue [29]. In this ex vivo study, vein segments were cultured and treated with Protandim, a botanical supplement reported to activate NRF2 through electrophilic modification of KEAP1 cysteine residues.

The authors found that Protandim treatment significantly increased the activity of three canonical NRF2 target enzymes: superoxide dismutase (SOD), catalase and HO-1. This enzymatic triad addresses different reactive species, as SOD converts superoxide to hydrogen peroxide, catalase detoxifies hydrogen peroxide to water and HO-1 degrades haem to produce the antioxidant biliverdin and carbon monoxide the vasodilator and anti-inflammatory mediator [30]. Critically, Protandim reduced tissue levels of superoxide and 4-HNE, directly connecting NRF2 activation to suppression of established IH mechanisms, with superoxide being a key activator of ERK1/2 MAPK signalling, driving proliferation and migration of VSMCs, and with 4-HNE serving as a signalling molecule that activates the ERK1/2, p38 MAPK and NFκB pathways [31].

Enhancing SOD activity would reduce the amount of superoxide available to activate MAPK, potentially interrupting the proliferative cascade driving IH. Activation of NRF2 would reduce 4-HNE levels and simultaneously suppress pro-proliferation and pro-inflammatory pathways. Moreover, NRF2 itself targets genes such as glutathione S-transferases and aldehyde dehydrogenases, which directly conjugate and eliminate 4-HNE, thereby creating a feedback mechanism that could prevent sustained MAPK activation.

Whilst mechanistically informative, this study [29] has critical limitations that affect its interpretation in this context. The study measured downstream enzyme activities without directly quantifying NRF2 nuclear translocation, KEAP1 modification or ARE binding. The increases seen in SOD, catalase and HO-1 are consistent with NRF2 activation but do not prove it. These enzymes can be regulated by alternative transcription factors such as activator protein 1, HIF-1α and heat-shock factor-1. Veins themselves were cultured without exposure to arterial shear stress or pulsatile flow. Since shear stress profoundly influences oxidative stress generation and NRF2 activation [32], these static culture findings may not accurately predict responses under in vivo conditions. The study did not measure any IH precursors such as intimal thickness or VSMC proliferation, so whether the observed antioxidant upregulation translates to a positive reduction or modulation of IH remains unknown. Similarly, interactions between NFκB, MAPK, eNOS, and angiogenic pathways were not examined, limiting understanding of how NRF2 activation might integrate with or antagonise other VGD mechanisms.

Despite these limitations, the Joddar study does provide foundational evidence that human long saphenous vein (LSV) tissue can mount an NRF2-like antioxidant response and that this specifically reduces oxidants known to drive VSMC proliferation via MAPK signalling.

### 4.2. The Biphasic Nature of NRF2 Activation in Vein Graft Disease

The mechanistic studies reviewed above reveal that NRF2 activation does not produce a uniform outcome in the vein graft setting. Its effect is biphasic and determined by the magnitude, duration, and cellular context of activation, as well as by the mechanism through which KEAP1 is inhibited.

In the acute phase of VGD development, which is the first 24–72 h after surgical harvest and implantation of the vein, the dominant stimulus is ischaemia–reperfusion injury, surgical trauma, and the abrupt transition from venous to arterial haemodynamics. In this context, ROS-mediated cysteine modification of KEAP1 produces transient NRF2 stabilisation [33]. This is the physiological mode of activation, and its consequences appear protective: direct ROS neutralisation via SOD, catalase, and HO-1 upregulation; suppression of NFκB-driven inflammation; preservation of eNOS coupling and NO bioavailability; and NOX4 transcriptional repression, reducing ongoing ROS generation at source. The net effect is a coordinated antioxidant response that limits endothelial apoptosis and suppresses the oxidant triggers of MAPK-dependent VSMC proliferation.

**Figure 2:**
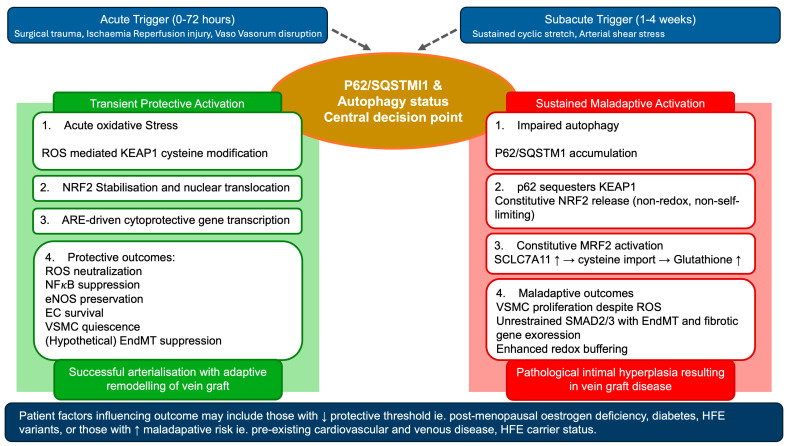
Biphasic model of NRF2 activation in saphenous vein grafts and its dependence on autophagy status. Two sequential haemodynamic triggers drive NRF2 activation after grafting: acute surgical trauma and ischaemia–reperfusion injury (0–72 h), followed by sustained arterial cyclic stretch during arterialisation (weeks 1–4). The functional outcome of NRF2 activation is determined by p62/SQSTM1 accumulation and autophagy status, which act as a central decision point. When autophagy is competent, transient ROS-mediated KEAP1 cysteine modification releases NRF2 in a self-limiting manner, driving ARE-dependent transcription of cytoprotective genes (HO-1, NQO1, SOD, catalase, GCLC/M, GCH1) and producing protective outcomes including ROS neutralisation, NFκB suppression, eNOS preservation, VSMC quiescence, and endothelial survival, culminating in successful arterialisation and graft patency. When autophagy is impaired, p62/SQSTM1 accumulates and sequesters KEAP1 via a distinct non-redox mechanism, producing constitutive NRF2 release that is non-self-limiting. This drives SLC7A11-mediated cystine import and glutathione accumulation, enabling VSMCs to tolerate proliferative oxidative stress while unrestrained SMAD2/3 signalling promotes EndMT and fibrotic gene expression, culminating in pathological intimal hyperplasia and vein graft failure. Patient factors that shift the threshold between protective and maladaptive states include post-menopausal oestrogen deficiency, diabetes, and HFE mutation (C282Y/H63D), which reduce the protective activation threshold, and pre-existing chronic venous disease or HFE carrier status, which predispose to the maladaptive state at the time of surgery. EndMT suppression by NRF2 is mechanistically proposed but has not yet been directly investigated in LSV.

During the early arterialisation phase, approximately one to four weeks following implantation, the vein begins to adapt to sustained arterial cyclic stretch. This mechanical environment impairs autophagy, causing accumulation of p62/SQSTM1 [34]. Unlike cysteine modification, p62-mediated KEAP1 sequestration is not self-limiting. It produces constitutive NRF2 activation that is decoupled from the redox state of the cell. In this context, enhanced glutathione synthesis and redox buffering paradoxically support VSMC survival under proliferative stress, promoting neointima formation rather than preventing it. This represents the maladaptive mode of NRF2 activation. This biphasic behaviour suggests that the transition from protective to maladaptive NRF2 activity may correspond to the shift from transient KEAP1 cysteine modification to sustained p62-dependent KEAP1 sequestration (Figure 2). The molecular threshold at which this transition occurs is unknown but likely varies by cell type, comorbidity profile and patient sex. In endothelial cells, where NRF2 activation suppresses NFκB and preserves eNOS function, the protective window may be broader. In VSMCs, where NRF2-enhanced redox buffering supports proliferative capacity, even moderate sustained activation may be maladaptive. This cell-type specificity has not been systematically examined in human saphenous vein and represents a critical mechanistic question for future investigation.

### 4.3. NRF2 Intersections with Established Inflammatory Intimal Hyperplasia Pathways

Whilst only the Joddar study has examined the NRF2/KEAP1 axis directly in human LSV tissue, there are many mechanistic studies, primarily in human umbilical venous endothelial cells (HUVECs) in vitro or in non-human animal tissues, which identify how NRF2 signalling intersects with pathways known to drive IH (summarised in Figure 1 and Supplementary Table S1). These connections are critical for beginning to define the potential role of NRF2 in VGD, although extrapolation from in vitro or non-human studies to the human saphenous vein in a coronary circulation requires caution.

#### 4.3.1. NRF2 and MAPK

p38 MAPK has been demonstrated to serve as an upstream activator of NRF2 in endothelial cells exposed to oxidative stress [35]. Treatment with willow bark extract activated p38, which promoted NRF2 nuclear translocation and ARE-driven transcription of HO-1, GCLM, and GCLC. Importantly, pharmacological inhibition of p38 abolished NRF2 activation, confirming p38 as an obligate intermediate. This creates a negative feedback loop whereby oxidative stress activates p38 MAPK, which activates NRF2, which produces antioxidants that suppress further p38 activation. However, in VSMCs, p38 also drives proliferation and migration. The balance between p38’s pro-proliferative and pro-NRF2 activities may therefore determine whether cells proliferate or activate antioxidant defence.

By modulating the downstream products of oxidative stress activation such as superoxide and 4-HNE [29], NRF2 activation would suppress ERK1/2 activation in VSMCs. Previous work [20] has demonstrated that ERK1/2 inhibition reduces intimal hyperplasia in vein grafts. Thus, NRF2 may indirectly suppress IH by removing oxidant triggers of ERK signalling.

A rat vein model has been used to identify the proliferative paradox of NRF2 [36]. Arterial cyclic stretch impaired autophagy, causing accumulation of p62/SQSTM1, KEAP1 sequestration and sustained activation of NRF2. This led to upregulation of the cystine transporter Solute Carrier Family 7 Member 11 (SLC7A11), enhanced glutathione synthesis, and increased VSMC survival and proliferation within the neointima and is discussed in detail in the biphasic activation section below. The key implication for the MAPK axis is that the relationship of NRF2 with VSMC proliferation is not uniformly suppressive. Transient NRF2 activation driven by acute ROS exposure removes MAPK activators such as superoxide and 4-HNE. However, sustained activation can create a redox-buffered environment where VSMC proliferation proceeds despite on-going oxidative signalling.

#### 4.3.2. NRF2 and NFκB

Multiple studies have shown that NRF2 and NFκB exhibit reciprocal inhibitory crosstalk. NRF2 and NFκB have been shown to compete for shared transcriptional coactivators, including CBP/p300 [37]. When NRF2 is active, it sequesters these coactivators, reducing NFκB-dependent transcription of inflammatory genes. This interaction has been demonstrated in the context of vein graft biology [32,38]. In HUVECs exposed to acute shear stress, mimicking the arterialisation process, NFκB activation drove expression of interleukin 8 (IL-8) and adhesion molecules. However, when NRF2 was overexpressed, the inflammatory response was attenuated. Importantly, the study showed that shear stress induced KEAP1 nuclear export, allowing for NRF2 accumulation and activation of HO-1 and GCLM. HO-1 is a key mechanism by which NRF2 suppresses inflammation. HO-1 generates carbon monoxide, which inhibits NFκB activation and reduces expression of adhesion molecules and cytokines [39]. HO-1 also produces biliverdin/bilirubin, potent antioxidants that neutralise oxidants that would otherwise activate NFκB. Deficiency of HO-1 is associated with accelerated vein graft failure, underscoring its importance [40]. The natural compound zedoarondiol activated NRF2 in HUVECs exposed to oxidised LDL, resulting in reduced expression of inflammatory cytokines IL-1β, TNF-α, MCP-1, and adhesion molecules ICAM-1 and VCAM-1 [41]. NRF2 activation suppressed NFκB p65 phosphorylation at Ser536, a critical step for NFκB nuclear translocation and transcriptional activity. When NRF2 was inhibited, the protective effects were abolished, confirming NRF2-dependency.

The NRF2-NFκB antagonism suggests that therapeutic NRF2 activation could simultaneously address two central drivers of IH: oxidative stress (through direct antioxidant gene induction) and inflammation (through NFκB suppression). This dual mechanism may be particularly important in vein grafts where oxidative stress and inflammation form a self-perpetuating cycle.

#### 4.3.3. NRF2 and iNOS

The inflammatory cascade triggered by NFκB activation in the grafted saphenous vein does not limit itself to cytokine and adhesion molecule expression. Among the most consequential downstream events is the transcriptional induction of inducible nitric oxide synthase (iNOS) [42]. In contrast to eNOS, which produces low, pulsatile amounts of NO under physiological signalling control, iNOS generates sustained, high-output NO independently of calcium regulation [43]. Within the grafted vein, NFκB activation occurs within 30 min of exposure to arterial shear stress in LSV ECs and drives a rapid pro-inflammatory programme, including chemokine CCL2 upregulation and monocyte recruitment. This response is prevented when the NFκB pathway is inhibited [38]. At the same time, NADPH oxidase generates large amounts of superoxide. When superoxide meets the high-output NO produced by iNOS, the two molecules react almost instantly to form peroxynitrite, a highly reactive compound which is strongly oxidising and which causes far more cellular damage than either molecule would cause on its own [44]. Peroxynitrite exerts irreversible damage across all three major classes of biological macromolecules. At the protein level, it nitrates tyrosine residues to form 3-nitrotyrosine, a stable post-translational modification detectable as a tissue biomarker of nitrosative stress. Nitrotyrosine formation has been detected in neointimal tissue in late-stage vascular disease, and its presence correlates with reduced soluble guanylyl cyclase activity and impaired cGMP signalling in the neointima, thereby compounding the eNOS-mediated vasoprotective loss [45]. Critically, peroxynitrite itself drives eNOS uncoupling and shifts eNOS output from NO to superoxide and so generates a positive feedback loop of oxidative and nitrosative injury [46]. At the lipid level, peroxynitrite initiates lipid peroxidation chain reactions producing oxidative stressors which alter the function of structural and signalling proteins [47]. At a nucleic acid level, peroxynitrite causes 8-nitroguanosine formation and single-strand DNA breaks, which activate the DNA damage sensor poly(ADP-ribose) polymerase 1 (PARP-1). Sustained PARP-1 activation depletes cellular NAD+ stores, compromising ATP generation and cell survival [48]. This pattern of protein nitration, lipid peroxidation, and DNA damage has been demonstrated in venous tissue under chronic inflammatory conditions [49]. These conditions share the NFκB-driven oxidative milieu characteristic of VGD. The NRF2/KEAP1 system can interrupt this damaging cycle at several points. NRF2 activation induces HO-1, which in turn suppresses iNOS expression and attenuates NFκB-driven inflammatory signalling. This reduces the volume of high-output NO available to react with superoxide and form peroxynitrite. NRF2 also restores the supply of tetrahydrobiopterin (BH4), the essential cofactor that maintains eNOS in its coupled, NO-producing configuration. When BH4 is depleted, eNOS uncouples and shifts its output from NO to superoxide, feeding directly into peroxynitrite production. NRF2 addresses this by upregulating GTP cyclohydrolase 1, which is the rate-limiting enzyme in BH4 synthesis, and by reducing the oxidative burden that usually results in conversion of BH4 to its inactive form. This preserved BH4 availability keeps eNOS coupled and restores NO output. By doing this, NRF2 interrupts one of the key amplification loops that sustain nitrosative injury [46,50]. Taken together, this positions the NRF2/KEAP1 axis as an upstream regulator of the broader nitrosative damage which perpetuates VGD pathology. Whether NRF2 activation is sufficient to produce a measurable reduction in nitrotyrosine burden or PARP activation in human LSV under arterial haemodynamic conditions has not been directly tested and remains an important question for future translational studies.

#### 4.3.4. NRF2 and eNOS

Oxidative stress reduces eNOS expression and uncouples the enzyme, causing it to produce superoxide instead of NO. The NRF2 activator pterostilbene has been shown to restore eNOS function in endothelial cells exposed to uraemic serum [51]. This restoration occurred alongside reduced ROS and increased antioxidant enzyme expression, suggesting that NRF2-mediated ROS reduction allows eNOS to function properly.

In a study of diabetic endothelial dysfunction, NRF2 activation directly represses NOX4 transcription, creating a negative feedback loop [52]. Histone deacetylase 3 (HDAC3) inhibition activated NRF2, which then suppressed NOX4 expression. This reduced ROS generation at the source. Conversely, silencing NOX4 increased NRF2 levels and its target genes (HO-1, NQO1, NQO2). This reciprocal regulation is crucial for VGD. NADPH oxidases are primary sources of ROS in vein grafts [22], and their upregulation by disturbed flow and inflammatory cytokines perpetuates oxidative injury. By suppressing NOX4, NRF2 activation would reduce the rate of ROS generation, not just scavenging ROS after they are produced. This represents a more sustainable antioxidant strategy. Under physiological conditions, NO suppresses NADPH oxidase activity. However, in oxidatively stressed vessels, superoxide scavenges NO to form peroxynitrite, reducing NO bioavailability and removing its tonic suppression of NADPH oxidase. NRF2 activation could break this cycle by reducing superoxide via SOD upregulation and suppressing NOX4 directly. NRF2 can therefore preserve bioavailability and restore the physiological balance between NO and ROS signalling.

Redox regulation is also provided by reactive sulfur species (RSS), particularly H_2_S and its oxidised derivatives, including polysulfides and nitrosopersulfide [53]. H_2_S is endogenously produced in vascular tissue by three enzymes: cystathionine gamma-lyase (CSE), cystathionine beta-synthase, and 3-mercaptopyruvate sulfurtransferase. CSE has been demonstrated to be expressed in the media, neointima, and intima of human saphenous vein segments, and its expression is negatively regulated by arterial shear stress [54]. CSE overexpression inhibits primary human VSMC migration without affecting proliferation, while its knockdown promotes migration, thus directly implicating endogenous H_2_S deficiency in IH development following arterialisation. This represents a flow-regulated RSS-dependent mechanism that compounds the oxidative and inflammatory burden on the grafted vein under arterial haemodynamic conditions.

The intersection of RSS and the KEAP1/NRF2 axis is mechanistically direct. H_2_S and polysulfides persulfidate cysteine residues on KEAP1, which are the same residues targeted by electrophilic NRF2 activators, stabilising NRF2 and driving ARE-dependent transcription of HO-1, NQO1, and glutathione synthesis enzymes [55]. Nitrosopersulfide is a bioactive product of the chemical interaction between H_2_S and NO and has been shown to activate Nrf2 nuclear accumulation and HO-1 mRNA expression in human vascular endothelial cells. It does so more potently than either parent molecule alone [56]. This positions RSS not as a separate pathway but as an upstream activator of KEAP1 cysteine modification and NRF2 stabilisation. H_2_S also directly augments eNOS activity and NO bioavailability. In models of vascular injury, a H_2_S donor has been shown to increase H_2_S production, enhance NRF2 nuclear accumulation, and upregulate HO-1 and superoxide dismutase with concomitant suppression of neointimal hyperplasia via NRF2/HIF-1α signalling [57].

The translational relevance of this is directly demonstrated in vein graft models. In murine vein grafts, local peri-procedural topical delivery of the H_2_S-releasing pro-drug GYY4137 has been shown to reduce intimal-to-medial area ratio by more than half and VSMC migration by almost a third [58]. Given that arterial shear stress downregulates CSE in LSV, reducing endogenous H_2_S at the moment of greatest haemodynamic and oxidative stress and supplementing RSS exogenously during harvest and implantation may compensate for this flow-driven deficit. Incorporating H_2_S-releasing compounds into vein preservation solutions could be a beneficial strategy in line with current attempts to modify preservation solutions to protect endothelial integrity during the harvest interval. Whether RSS supplementation and KEAP1-targeted NRF2 activation act additively or synergistically in this context warrants experimental investigation in human saphenous vein tissue.

#### 4.3.5. NRF2 and Endothelial Protection

Early endothelial cell death is a critical event in vein graft disease, exposing the thrombogenic basement membrane and initiating inflammatory cascades [59]. It is apparent that NRF2 activation protects against this through several mechanisms. Long non-coding RNA MALAT1 activates NRF2 signalling and protects endothelial cells from oxidative-stress-induced apoptosis [60]. MALAT1 overexpression reduced KEAP1 mRNA, increased NRF2 and its target genes (HO-1, NQO1, GCLC), and reduced apoptosis in response to hydrogen peroxide. Conversely, MALAT1 knockdown enhanced apoptosis and suppressed NRF2 signalling. Similar findings were demonstrated with sulodexide, showing that NRF2 activation reduced pro-apoptotic markers Bax, cleaved caspase-3, and established a strong negative correlation between ROS production and cell viability (R^2^ = 0.78, *p* < 0.001) [61]. By suppressing oxidative-stress-induced apoptosis, NRF2 activation could maintain endothelial integrity, preserve NO production and prevent the cascade of events leading to IH, thereby representing a primary prevention mechanism rather than a treatment for established disease.

#### 4.3.6. NRF2 and Angiogenic Regulation

Beyond direct cryoprotection, NRF2 also regulates angiogenesis in ways directly relevant to vein graft biology. Under ischaemic conditions, NRF2 supported physiological angiogenesis and endothelial cell survival while simultaneously suppressing pathological neovascularisation more characteristic of tumour angiogenesis [23]. In the context of vein grafts, control of neovascularisation is important. Immediately after grafting, a degree of angiogenesis is necessary to restore perfusion of the vein wall after vasa vasorum disruption during vein harvesting. However, excessive neovascularisation delivers inflammatory cells and growth factors to the intima and promotes IH. NRF2’s ability to promote survival-supporting angiogenesis while restraining pathological vessel growth could represent an optimal balance, though this has not been tested in vein graft models. This angiogenesis is largely regulated by HIF-1α, and NRF2 activation can indirectly reduce HIF-1α stabilisation, potentially tempering excessive angiogenic responses. Rutin quinone, an NRF2 activator, has been shown to simultaneously enhance NRF2 transcriptional activity while blunting HIF activation, supporting this regulatory interaction [62].

#### 4.3.7. Mechanical Forces and NRF2 Protection

Mechanical forces exerted upon arterialised vein grafts provide another dimension to NRF2 regulation. Acute arterial shear stress (12 dyn/cm^2^ for 30 min) induced KEAP1 nuclear export in HUVECs without immediately increasing nuclear NRF2 [63]. Despite this, shear stress activated NRF2-regulated genes, including HO-1, GCLM, and IL-8. This suggests that shear stress modulates the KEAP1-NRF2 system through subcellular redistribution mechanisms that may differ from classical oxidant-induced KEAP1 modification. When KEAP1 was knocked down (via sulforaphane treatment or siRNA), both HO-1 and IL-8 expression increased further under both static and shear conditions. Importantly, a dominant-negative NRF2 mutant abolished HO-1 induction but did not suppress IL-8, indicating that while NRF2 is necessary for the antioxidant response to shear, inflammatory gene activation involves additional NRF2-independent pathways.

When a saphenous vein is grafted into coronary circulation, it experiences an abrupt increase from venous shear stress (~1–5 dyn/cm^2^) to arterial shear stress (~10–20 dyn/cm^2^). This mechanical stress would trigger KEAP1 redistribution and NRF2 activation, potentially explaining why some vein grafts successfully adapt while others develop IH. Veins with robust NRF2 responses might mount sufficient antioxidant defence to prevent progression to pathological remodelling. Arterial shear stress not only activates this protective NRF2 signalling but also appears to trigger a maladaptive response that can overwhelm these defences. Earlier work in our group demonstrated that acute exposure of venous endothelial cells to arterial shear stress induces endothelial-to-mesenchymal transition (EndMT) via the transforming growth factor-beta (TGFβ) and small mothers against decapentaplegic (SMAD) pathway via TWIST 1 and 2 transcription factors [64]. EndMT is characterised by a downregulation of endothelial markers such as VE-cadherin in favour of upregulation of mesenchymal marks such as vimentin, representing a shift in phenotype that contributes to formation of a neointima. Using spatial transcriptomics on human saphenous vein segments from patients undergoing coronary bypass grafting, it was possible to identify a sub-population of cells demonstrating these hybrid endothelial and smooth muscle cell characteristics. This subpopulation exhibits significant upregulation of TWIST2, suggesting active transitional remodelling. The balance between protective NRF2 activation and deleterious EndMT activation may therefore determine whether a vein graft can successfully integrate into the arterial system or whether it will develop pathological intimal hyperplasia under the same stimuli.

Beyond the acute transition from venous to arterial shear stress at the time of implantation, the grafted vein is subsequently exposed to a varied haemodynamic environment where the balance between laminar and oscillatory flow determines both the local inflammation and the capacity for NRF2-mediated protection. In chronic venous disease, saphenous vein incompetence generates oscillatory, non-laminar flow at the saphenofemoral junction. This oscillatory flow drives a proinflammatory cytokine profile in the vein wall, with elevated IL-1β, interferon-gamma, interleukins 2, 4, 8 and 12, and MCP-1 detectable in incompetent LSV compared to veins exhibiting laminar flow in the same patients [65]. Critically, when laminar flow is restored after surgical correction there is a significant reduction in the cytokine burden, demonstrating that the inflammatory phenotype of the LSV in some environments is flow-dependent and at least partially reversible [66]. In the coronary grafted vein, there remain areas where flow is persistently disturbed, such as at sites of anastomosis. These areas of turbulent flow sustain NOX-mediated ROS generation, activating AKT/BIRC5 survival signalling in VSMCs, and drive VSMC proliferation, migration, and resistance to apoptosis. This mechanism has been directly demonstrated in vein graft models exposed to high oscillatory shear stress [67]. Comparatively, areas of laminar flow may exert a counter-regulatory effect through NRF2. Prolonged steady laminar shear stress has been demonstrated to activate ARE-mediated transcription of protective products NQO1, HO-1, ferritin, and glutathione S-transferases via NRF2 in endothelial cells [68]. The subsequent NRF2 expression supresses TNF-α-induced VCAM-1 expression, defining a mechanosensitive, NRF2-dependent anti-inflammatory pathway [14]. This suggests that the vein graft does not experience a uniform stimulus after implantation but that the oxidative and inflammatory stresses are experienced variably within the vein with laminar flow, perhaps activating protective NRF2 signalling, while disturbed flow may sustain oxidative injury and NFκB-driven inflammation [15]. Whether NRF2 activation in laminar-flow regions is sufficient to provide paracrine protection to adjacent regions under oscillatory stress is unknown. This variation across different regions of the vein may partly explain why IH in vein grafts tend to be focal and why lesions cluster at anastomoses. This underscores the importance of surgical technique when composing grafts given their role as determinants of the local NRF2 and oxidative signalling environment.

#### 4.3.8. NRF2 and Endothelial-to-Mesenchymal Transition

Notably, NRF2 has been shown to antagonise TGFβ signalling in other contexts of vascular disease [69,70], raising the possibility that robust NRF2 activation may supress EndMT in veins. This has not been investigated to our knowledge and would be an interesting potential mechanism which could be targeted for future therapeutic development in the context of intimal hyperplasia propagation. Understanding how NRF2 might antagonise TGFβ/SMAD-mediated EndMT requires examination of the molecular crosstalk between these pathways.

The observation that arterial shear stress simultaneously activates both NRF2 signalling (via KEAP1 redistribution) and TGFβ/SMAD-mediated EndMT raises the question of how these pathways interact in the context of vein grafts and their resultant disease. Emerging evidence suggests a reciprocal inhibitory relationship between KEAP1/NRF2 axis and TGFβ/SMAD signalling, suggesting that NRF2 could represent a therapeutic strategy to attenuate TGFβ-driven EndMT in vein grafts. Stable KEAP1 knockdown (resulting in constitutive NRF2 expression) supresses TGFβ-stimulated SMAD2/3 phosphorylation and the expression of pro-fibrotic genes encoding fibronectin-1 and collagen 1A1.

A new mechanism was observed whereby KEAP1 physically interacts with SMAD2/3 through conserved motifs homologous to those used by NRF2, creating a direct molecular bridge whereby NRF2 and SMAD2/3 may compete for KEAP1 binding [71]. This results in KEAP1 directly sequestering SMAD2/3 in the cytoplasm, providing an additional mechanism by which the KEAP1/NRF2 axis may modulate TGFβ signalling. Consistent with these findings, studies in models of intestinal fibrosis have demonstrated that NRF2 prevents the transformation of fibroblasts to myofibroblasts by inhibiting the ROS-dependent TGFβ/SMAD pathway, whilst NRF2 agonists effectively supress TGFβ levels and downstream fibrotic gene expression [72] (Figure 1). Furthermore, NRF2/HO-1 activation preserves endothelial barrier integrity by enhancing tight junction protein expression through the ERK/NRF2/HO-1 signalling cascade [73], and the products of HO-1-mediated haem degradation then exert additional anti-inflammatory and anti-apoptotic effects that can further protect from the chronic inflammatory environment, driving vascular remodelling. Given that TGFβ/SMAD-driven EndMT contributes to the development of intimal hyperplasia and vein graft failure through the acquisition of a mesenchymal, profibrotic phenotype of endothelial cells, pharmacological activation of NRF2 may offer a dual benefit in VGD: it may enhance antioxidant defence against oxidative stress and simultaneously supress EndMT onset that can drive pathological remodelling. Taken together, the mechanistic evidence reviewed above defines a hierarchical structured network where KEAP1 occupancy functions as the central node. The availability of KEAP1 determines both the stability of NRF2 and the cytoplasmic retention of SMAD2/3. When KEAP1 cysteine residues are transiently modified by ROS, NRF2 is stabilised and SMAD2/3 are released to compete for the same binding domain [71]. Under these conditions, NRF2 nuclear activity suppresses NFκB-driven inflammation, restores eNOS coupling, reduces NOX4-dependent ROS generation, and limits the oxidant triggers of ERK1/2 and p38 MAPK activation in VSMCs. Simultaneously, partial SMAD2/3 release may initiate early adaptive remodelling. When KEAP1 is instead sequestered by p62 because of autophagy dysfunction, constitutional activation of NRF2 enhances redox buffering capacity in VSMCs. This supports proliferation, while unrestrained SMAD2/3 signalling promotes EndMT and fibrotic gene expression [34,71]. The balance between these two states is determined by the mechanism of KEAP1 inhibition rather than simply the level of NRF2 activity. Therefore, it determines whether the vein graft undergoes successful adaptive arterialisation or progresses to pathological IH. This positions KEAP1 not merely as a negative regulator of NRF2 but as a molecular switch integrating oxidative, proliferative, and mesenchymal signals in the vein grafts.

## 5. Inter-Patient Variability and Its Influence on the Development of VGD

The evidence reviewed above describes a vascular environment a in which the mode, magnitude, and duration of KEAP1 inhibition determines clinical outcome rather than simply the level of NRF2 activity (Figure 2). Transient cysteine modification by ROS activates a coordinated protective programme: direct scavenging of superoxide and hydrogen peroxide removes the oxidant triggers of MAPK-driven VSMC proliferation; NRF2-NFκB antagonism suppresses cytokine and adhesion molecule expression; eNOS recoupling preserves NO bioavailability and VSMC quiescence; NOX4 transcriptional repression reduces ongoing ROS generation at source; and anti-apoptotic signalling preserves endothelial integrity. Each of these effects corresponds to a specific, targetable therapeutic opportunity during the peri-operative period. The transition to maladaptive NRF2 activity, driven by p62-mediated KEAP1 sequestration under sustained cyclic stretch, does not generate these same protective outputs. Instead, it produces constitutive redox buffering that supports VSMC proliferation. This distinction has direct implications for the timing and delivery strategy of any NRF2-targeted intervention, as discussed in the Section 6 below. Patients with suppressed NRF2 function due to diabetes, HFE genotype, or post-menopausal oestrogen deficiency may sit below the protective threshold, while patients with pre-existing chronic venous disease may already occupy the maladaptive state at the time of surgery. Stratifying patients by these variables before applying NRF2-targeted therapy may be as important as the choice of therapeutic agent.

Inter-patient variability in NRF2 pathway function is likely to be clinically significant, and biological sex represents one dimension of this that warrants specific attention. Clinical data consistently demonstrate that women experience worse vein graft outcomes following CABG than men, yet the molecular mechanisms underlying this disparity remain poorly understood [74]. No direct evidence from human LSV currently links sex-specific biology to NRF2/KEAP1 pathway function. The following therefore represents a mechanistic hypothesis, grounded where possible in indirect vascular evidence and intended to frame a research agenda for future work by extrapolation of related work but not to summarise established sex-specific findings.

Oestrogen enhances eNOS expression and NO bioavailability through well-characterised genomic and non-genomic mechanisms in vascular endothelial cells [75]. Since NRF2 modulates both eNOS coupling and NOX4 expression, and since most female CABG patients are post-menopausal with reduced oestrogen levels, the loss of oestrogen-mediated support for the NO-ROS balance could alter both the threshold at which NRF2 activation is required and the adequacy of the resulting response. Women generally exhibit higher baseline levels of pro-inflammatory cytokines than men across multiple vascular contexts [76]. Given the reciprocal inhibitory relationship between NRF2 and NFκB, a higher constitutive inflammatory drive could reduce the effective anti-inflammatory benefit of equivalent NRF2 activation in female vein tissue. Sex hormones profoundly influence histone modification patterns. HDAC3 activity suppresses NRF2 by upregulating KEAP1, as demonstrated in vascular ECs [52]. In addition, methyltransferase SET8 has been demonstrated to maintain KEAP1 expression through histone H4K20 monomethylation, and SET8 is itself subject to hormonal regulation [77]. Post-menopausal changes in epigenetic state could therefore enhance both HDAC3 and SET8 activity, reducing NRF2 responses at precisely the moment when oxidative burden increases. Furthermore, oestrogen can directly activate NRF2 through non-genomic mechanisms, and its loss removes this additional activation pathway [78]. Women also exhibit sex-specific expression of aldehyde dehydrogenases and glutathione S-transferases, which are NRF2 target genes responsible for 4-HNE elimination. If baseline expression differs, the same degree of NRF2 activation might produce different 4-HNE elimination rates, resulting in sex-specific MAPK activation patterns even with equivalent antioxidant responses.

Recent work has identified X-chromosome-linked microRNAs that regulate cardiac physiology in sex-specific ways [79]. Women possess two X chromosomes, with incomplete X-inactivation meaning some regulatory genes exhibit higher expression. If X-linked genes encode KEAP1 regulators or NRF2 suppressors, women might require greater oxidative stress to achieve equivalent NRF2 activation. The interaction between these sex-specific mechanisms and the p62-mediated maladaptive NRF2 activation described above has not been explored.

To begin addressing this gap, three specific research directions are proposed. First, direct comparison of NRF2 nuclear translocation, KEAP1 expression, and ARE-binding activity in male and female human saphenous vein tissue under standardised ex vivo conditions would establish whether a baseline sex difference in pathway activity exists. Second, patient-stratified studies prospectively correlating NRF2 pathway activation in harvested vein with graft patency outcomes, analysed separately by sex, would determine whether this difference has clinical significance. Third, examination of oestrogen receptor signalling on KEAP1 promoter activity in venous endothelial cells would provide a direct test of the proposed hormonal mechanism.

Another relevant source of inter-patient variability in baseline vein wall oxidative burden is HFE genotype. C282Y and H63D mutations in the HFE gene are common in Northern European populations and impair hepcidin-mediated iron regulation, which results in iron accumulation [80]. In the context of chronic venous disease, red blood cell extravasation driven by venous hypertension deposits hemosiderin in the local tissues [81]. In HFE mutation carriers, macrophage intracellular iron stores are less stable than wild-type ones, releasing free iron that generates ROS locally. C282Y mutation increases the risk of venous leg ulceration by nearly sevenfold in primary chronic venous disease [82], with H63D carriers demonstrating ulcer onset approximately a decade earlier than non-carriers [83]. These findings have been independently confirmed in a separate population [84]. These mutations are highly relevant to patients undergoing CABG using venous conduits. A saphenous vein harvested from a patient with a HFE mutation may already carry pre-existing endothelial dysfunction, perivenous iron deposition, and chronically elevated levels of ROS prior to its exposure to the additional oxidative insults of surgical harvest, ischaemia, and arterialisation. If p62-mediated KEAP1 sequestration is already occurring in the vein wall because of sustained venous oxidative stress, NRF2 may be constitutively activated at baseline. As discussed above in the context of a rat vein graft model [34], this represents the maladaptive state in which sustained NRF2 activation promotes VSMC proliferation through enhanced glutathione synthesis and redox buffering. As such, the vein graft would therefore enter the coronary circulation with a depleted ability to adapt and may be unable to mount a proportionate protective NRF2 response to the subsequent acute haemodynamic and ischaemic insults. HFE genotyping combined with pre-operative assessment of venous disease severity may be considered as variables in future mechanistic studies of NRF2 pathway activation in human saphenous vein and may inform patient-specific peri-operative antioxidant strategies.

## 6. Translational Implications

The mechanistic connections between NRF2 and established VGD pathways suggest multiple therapeutic opportunities, though each approach carries distinct advantages and limitations that must be carefully considered. The most promising strategy appears to be direct KEAP1 inhibition through cysteine modification. Two studies demonstrated that targeting Cys151 on KEAP1 effectively activates NRF2 without altering gene transcription [62,85]. Compounds like withaferin A and rutin quinone used in these studies modify this residue, stabilise NRF2, and induce robust antioxidant responses. This post-translational mechanism offers the advantage of avoiding any transcriptomic off-target effects, reversible modification allows transient activation, and it would simultaneously affect multiple downstream pathways, including MAPK suppression, NFκB inhibition, and eNOS preservation. Potential delivery strategies include ex vivo treatment of vein segments before implantation by incorporating it into preservation solutions during harvest [7], perivascular delivery systems releasing activators locally post-implantation [86], or systemic administration peri-operatively during peak oxidative stress.

Alternative approaches targeting upstream regulatory mechanisms warrant consideration, particularly for specific patient populations. HDAC3 inhibition represents one such strategy that upregulates NRF2 by reducing KEAP1 expression. This might be particularly valuable in diabetic patients where HDAC3 activity is elevated and may contribute to suppressed antioxidant responses. However, HDAC inhibitors have broad effects beyond KEAP1-NRF2, requiring careful safety assessment to ensure benefits outweigh risks. Similarly, since p38 MAPK acts upstream of both NRF2 activation and VSMC proliferation [87], selective p38 modulation could theoretically enhance the protective arm while suppressing the proliferative arm. However, this would require isoform-selective inhibitors and careful timing to avoid blocking the p38-NRF2 protective axis while inhibiting p38-driven proliferation.

Given NRF2’s integration with multiple pathways, combination strategies might prove most effective for comprehensive vein graft protection. An NRF2 activator combined with an NFκB inhibitor could synergistically suppress inflammation through reciprocal mechanisms, addressing both oxidative and inflammatory axes simultaneously. Combining NRF2 activators with NO donors could address both oxidative stress and eNOS dysfunction, potentially restoring the physiological balance between NO signalling and ROS generation. Additionally, combining NRF2 activators with statins presents an attractive option since statins reduce NADPH oxidase expression and when combined with NRF2-mediated NOX4 suppression, and this could achieve maximal source control of ROS generation while simultaneously enhancing antioxidant defences.

The timing of therapeutic intervention may be as important as the choice of agent (Figure 2). Based on the findings showing maladaptive NRF2 effects under chronic arterial stretch, optimal therapy likely involves a pulsed approach rather than sustained activation. Pre-operative or peri-operative activation could prime antioxidant defences before and during peak acute oxidative stress, when the vein experiences surgical trauma, ischaemia, and initial exposure to arterial haemodynamics. Early post-operative maintenance during the first two to four weeks would continue moderate activation during the critical arterialisation period when adaptive remodelling occurs. However, subsequent withdrawal or transition to pulsed dosing would be important to avoid sustained activation that might enhance proliferative capacity through p62-mediated mechanisms. This temporal strategy would harness NRF2’s protective effects during vulnerable periods while avoiding potential maladaptive consequences of chronic activation that could paradoxically promote intimal hyperplasia.

## 7. Conclusions

The KEAP1/NRF2 signalling pathway functions as a central integration point for oxidative stress responses that directly intersect with multiple established mechanisms of vein graft disease including MAPK-driven proliferation, NFκB-mediated inflammation, eNOS-NO dysfunction, NADPH oxidase-generated ROS, and angiogenic dysregulation. The single study directly examining NRF2 pathway modulation in human saphenous vein tissue demonstrated that activation increases antioxidant enzyme activity and reduces superoxide and 4-HNE, both of which are key drivers of MAPK-dependent smooth muscle proliferation. However, this foundational evidence was generated in static culture without direct measurement of NRF2 signalling or assessment of proliferation, leaving critical questions unanswered. Mechanistic studies in endothelial cell models reveal that NRF2 activation can suppress multiple pathological processes: it reduces ROS availability for MAPK activation, antagonises NFκB inflammatory signalling, preserves eNOS function, directly suppresses NADPH oxidase transcription, and prevents oxidative-stress-induced apoptosis. These interconnected effects position NRF2 as a potential master regulator capable of interrupting the vicious cycle of oxidative stress, inflammation, and proliferation that drives intimal hyperplasia. However, the pathway exhibits context-dependent duality. While transient activation appears protective, sustained NRF2 activity driven by impaired autophagy can paradoxically enhance VSMC proliferation through increased redox buffering capacity. This finding from rat vein grafts emphasises that therapeutic NRF2 modulation requires careful consideration of timing, magnitude, cellular context, and haemodynamic environment.

Sex-specific differences in vein graft outcomes, combined with known effects of hormones and X-chromosome-linked genes on redox biology, suggest that NRF2 pathway function may differ fundamentally between male and female patients. Post-menopausal women might exhibit suppressed NRF2 responses due to epigenetic mechanisms while simultaneously showing enhanced inflammatory drive, making then vulnerable to accelerated IH. This dimension has been almost entirely neglected in existing research.

The most promising therapeutic approach appears to be post-translational KEAP1 inhibition through cysteine modification, which may allow for transient NRF2 stabilisation without broad transcriptomic changes. Delivery via ex vivo treatment to the vein through modified preservation solutions or perivascular systems during surgery could provide targeted intervention during the critical peri-operative period when oxidative stress peaks. Combined with NFκB inhibitors or NO donors, this might provide synergistic effects addressing multiple pathological concerns simultaneously.

Direct mechanistic characterisation of NRF2/KEAP1 signalling in human saphenous vein tissue under physiologically relevant conditions is an urgent research priority. This requires ex vivo organ culture models that expose human LSV segments to arterial-level pulsatile shear stress, with direct quantification of NRF2 nuclear translocation, KEAP1 modification, and ARE-binding activity, rather than relying on downstream enzyme activity as a surrogate. Patient-stratified biobanking studies prospectively correlating NRF2 pathway activation in harvested vein with graft patency outcomes would establish whether baseline NRF2 activity predicts clinical failure. These studies should stratify by sex, diabetic status, and HFE genotype, given the mechanistic reasons outlined above to expect that NRF2 function differs across these groups, and they should analyse male and female tissue separately to begin addressing the sex-specific gap that currently prevents meaningful translation to the CABG population. Whether a baseline sex difference in pathway activity exists could be established through direct comparison of NRF2 nuclear translocation, KEAP1 expression, and ARE-binding activity in male and female tissue under standardised ex vivo conditions, with examination of oestrogen receptor signalling on KEAP1 promoter activity in venous endothelial cells providing a direct test of the proposed hormonal mechanism. The interaction between NRF2 activation and EndMT in human saphenous vein has not been examined and represents a tractable experimental question with direct therapeutic implications. Finally, the proposed synergy between RSS supplementation and KEAP1-targeted NRF2 activation should be tested in human LSV organ culture before any translational claims are made. Fifteen years after the Joddar study established a proof of concept that human saphenous vein can mount an NRF2-like antioxidant response, the field remains at the threshold of understanding. The mechanistic connections between NRF2 and established VGD pathways are compelling, but we have a significant disconnect between our current in vitro models used to generate evidence and the actual biology of adult human saphenous vein under arterial shear stress. Direct, rigorous investigation in the relevant tissue under relevant conditions is essential to determine whether NRF2 pathway modulation represents a viable strategy for preventing vein graft failure or whether the complexity and context-dependency of this system limit its therapeutic potential.

## Data Availability

No new data were created or analyzed in this study.

## Supplementary Materials

The following supporting information can be downloaded at: https://www.mdpi.com/article/10.3390/cells15060563/s1, Table S1: Summary of Mechanistic Studies Cited in This Review.
