# Supplementary material for: Oxidative Stress and the KEAP1/NRF2 Axis in Saphenous Vein: Implications for Graft Patency
Source: Cells. 2026 Mar 20;15(6):563. doi: 10.3390/cells15060563 (PMC13025489; doi:10.3390/cells15060563)
Supplement: Supplementary file 1 [file cells-15-00563-s001.zip › cells-4158982-supplementary.pdf]

Supplementary Table 1. Summary of Mechanistic Studies Cited in This Review

Studies are ordered by thematic section as they appear in the manuscript. All provide mechanistic insight that is complimentary to NRF2/KEAP1 signalling and its intersection with vein graft disease pathobiology. Readers should maintain awareness that not all studies directly evaluate the NRF2/KEAP1 axis and therefore interpretation of these studies within the review are sometimes extrapolated into the context of vein graft disease but have not yet been evaluated directly in experimental study. Ref = manuscript reference number.

Ref	Study (First Author, Year)	Model System	Intervention / Pathway Examined	Key Mechanistic Finding	Relevance to VGD / IH Pathway
**Direct Human LSV Evidence**					
**29**	**Joddar et al., 2011**	Human saphenous vein segments — ex vivo organ culture (static)	Protandim (botanical NRF2 activator; electrophilic KEAP1 cysteine modification)	NRF2 activation increased SOD, catalase, and HO-1 activity; directly reduced tissue superoxide and 4-HNE. Only study directly examining NRF2-like antioxidant response in human LSV.	MAPK: superoxide and 4-HNE are key ERK1/2 and p38 activators driving VSMC proliferation. NRF2 activation removed both oxidant triggers, potentially interrupting the IH proliferative cascade.
**54**	**Zhao et al., 2023**	Human saphenous vein segments and primary human VSMCs	CSE overexpression and knockdown; arterial shear stress exposure (flow chamber)	CSE expressed in LSV media, neointima, and intima. Arterial shear downregulates CSE. CSE overexpression inhibits VSMC migration; knockdown promotes migration.	RSS/H2S: arterial shear suppresses endogenous H2S at peak haemodynamic stress, implicating RSS deficiency in post-arterialisation IH and removing a key NRF2-activating KEAP1 persulfidation input.
**64**	**Ladak et al., 2025**	Human LSV endothelial cells and vein segments ex vivo; spatial transcriptomics on human CABG tissue	Acute arterial shear stress; TWIST1/2 and TGFβ/SMAD pathway analysis	Arterial shear induces TWIST1/2-mediated EndMT via TGFβ/SMAD in venous ECs. Spatial transcriptomics identified a TWIST2+ hybrid EC-VSMC subpopulation in human CABG vein.	EndMT: directly demonstrates that arterial haemodynamics trigger mesenchymal transition in human LSV — the same stimulus that activates NRF2 via KEAP1 redistribution, establishing competing simultaneous pathway activation.
**Venous ECs Under Haemodynamic Stress**					
**32**	**Ward et al., 2022**	HUVECs under acute arterial shear stress (12 dyn/cm²)	NRF2 overexpression; KEAP1 knockdown (sulforaphane and siRNA)	Shear induced KEAP1 nuclear export and NRF2 accumulation; upregulated HO-1 and GCLM. NFkB drove IL-8 and adhesion molecules; NRF2 overexpression attenuated the inflammatory response.	NFkB/NRF2 crosstalk: competing activation of both pathways under arterialisation-mimicking shear, directly relevant to the balance of protective vs inflammatory responses during vein grafting.
**38**	**Ward et al., 2020**	Human saphenous vein ECs and LSV segments ex vivo under acute shear stress	NFkB pharmacological inhibition	NFkB inhibition prevented acute shear-induced IL-8, CCL2, and adhesion molecule expression in human LSV endothelium. NFkB activated within 30 minutes of arterial shear exposure.	NFkB cascade: establishes the rapid NFkB-driven pro-inflammatory programme in human LSV ECs on arterialisation — the cascade that NRF2-NFkB antagonism may therapeutically counteract.
**63**	**Ward, 2018 [Doctoral Thesis]**	HUVECs under acute arterial shear stress	Dominant-negative NRF2 mutant; KEAP1 knockdown (sulforaphane and siRNA)	Shear caused KEAP1 nuclear export without immediately increasing nuclear NRF2. Dominant-negative NRF2 abolished HO-1 but not IL-8, separating NRF2-dependent	Haemodynamic stress: defines the subcellular redistribution mechanism by which arterial shear activates KEAP1-NRF2 in venous ECs, and establishes parallel NRF2-independent

Ref	Study (First Author, Year)	Model System	Intervention / Pathway Examined	Key Mechanistic Finding	Relevance to VGD / IH Pathway
				antioxidant from NRF2-independent inflammatory activation.	inflammatory activation that also requires therapeutic targeting.
**68**	**Chen XL et al., 2003**	Human aortic ECs under prolonged steady laminar shear stress (15 dyn/cm2)	Laminar shear; ARE-reporter and gene expression assays	Laminar flow activated ARE-mediated transcription of NQO1, HO-1, ferritin, and glutathione S-transferases via NRF2. NRF2 expression subsequently suppressed TNF-a-induced VCAM-1.	Mechanosensitive anti-inflammatory pathway: laminar flow regions in vein grafts may sustain protective NRF2 signalling, contrasting with disturbed oscillatory flow at anastomoses where NFkB predominates.
**15**	**Dai et al., 2007**	Human aortic ECs; atherosclerosis-resistant vascular regions in vivo	PI3K/Akt pathway inhibition under laminar shear stress	Biomechanical forces in atherosclerosis-resistant vascular regions activate NRF2 via PI3K/Akt-dependent phosphorylation, maintaining endothelial redox balance.	Mechanical forces: PI3K/Akt-NRF2 mechanosensitive activation in laminar flow regions may provide sustained low-level cytoprotection in vein grafts, distinct from acute KEAP1 redistribution under shear.
**NRF2 and NFkB**					
**37**	**Nair et al., 2008**	In vitro cell lines; transcriptional coactivator competition assays	NRF2 and NFkB competitive binding analysis for CBP/p300 coactivators	NRF2 and NFkB compete for shared CBP/p300 transcriptional coactivators. Active NRF2 sequesters coactivators, directly reducing NFkB-dependent inflammatory gene transcription.	NFkB crosstalk: provides the molecular mechanism for NRF2-NFkB reciprocal inhibition — explains how a single NRF2 activation event simultaneously suppresses oxidative stress and inflammation, two co-drivers of IH.
**41**	**Mao et al., 2018**	HUVECs exposed to oxidised LDL	Zedoarondiol (natural compound NRF2 activator); NRF2 siRNA inhibition	Zedoarondiol activated NRF2 and reduced IL-1b, TNF-a, MCP-1, ICAM-1, and VCAM-1. Suppressed NFkB p65 phosphorylation at Ser536. All effects abolished by NRF2 inhibition, confirming NRF2-dependence.	NFkB cascade: NRF2-dependent suppression of the full inflammatory mediator profile relevant to vein graft endothelial activation and leucocyte recruitment driving IH.
**14**	**Zakkar et al., 2009**	Porcine aortic ECs; atherosclerosis-resistant vascular regions in vivo	NRF2 activation via laminar flow and sulforaphane	NRF2 activation protected endothelial cells from proinflammatory state, reducing adhesion molecule expression and leucocyte binding under inflammatory stimulation.	NFkB/endothelial protection: establishes endothelial NRF2 activation suppresses the proinflammatory adhesion molecule programme directly relevant to early vein graft endothelial activation.
**NRF2 and MAPK**					
**35**	**Ishikado et al., 2013**	HUVECs and Caenorhabditis elegans	Willow bark extract; pharmacological p38 MAPK inhibition (SB203580)	p38 MAPK acts as upstream activator of NRF2 in ECs under oxidative stress. p38 activation promoted NRF2 nuclear translocation and ARE-driven HO-1, GCLM, GCLC. p38 inhibition abolished NRF2 activation.	MAPK/NRF2 negative feedback: oxidative stress activates p38-NRF2, producing antioxidants that reduce oxidant triggers of further p38 activation. In VSMCs, p38 also drives proliferation — creating a therapeutically relevant balance between protective and proliferative signals.

Ref	Study (First Author, Year)	Model System	Intervention / Pathway Examined	Key Mechanistic Finding	Relevance to VGD / IH Pathway
17	**Ashino et al., 2016**	VSMCs; murine femoral artery wire injury model in vivo	NRF2/KEAP1 pathway modulation; VSMC apoptosis and proliferation assays	NRF2/KEAP1 regulates VSMC apoptosis for vascular homeostasis. NRF2 has context-dependent, potentially maladaptive roles in VSMCs and neointimal formation — distinct from its protective role in ECs.	VSMC biology: confirms NRF2 has cell-type-specific effects in VSMCs, directly supporting the cell-type specificity component of the biphasic model and cautioning against sustained NRF2 activation in the VSMC compartment.
**Biphasic Model / p62-KEAP1 Sequestration**					
34	**Chen et al., 2022**	Rat vein graft model; VSMCs under arterial cyclic stretch in vitro	Autophagy modulation; p62/SQSTM1 and SLC7A11 pathway analysis	Arterial cyclic stretch impaired autophagy, causing p62/SQSTM1 accumulation and KEAP1 sequestration. Constitutive NRF2 activation upregulated SLC7A11, enhanced glutathione synthesis, and promoted VSMC survival and neointimal proliferation.	Biphasic model (central study): demonstrates that the mode of KEAP1 inhibition determines whether NRF2 is protective or drives IH. Foundation of the maladaptive arm of Figure 2 — p62-mediated constitutive activation vs transient cysteine modification.
28	**Kageyama et al., 2018**	Cell lines; biochemical p62/SQSTM1 binding domain assays	p62/SQSTM1 splicing variant overexpression; KEAP1 binding domain analysis	p62/SQSTM1 splicing variant sequesters KEAP1, preventing NRF2 ubiquitination. Produces constitutive, non-self-limiting NRF2 activation independent of cellular redox state.	Biphasic model: defines the molecular mechanism of p62-mediated constitutive NRF2 activation — the non-redox pathway underlying the maladaptive phase when autophagy is impaired under sustained arterial cyclic stretch.
**NRF2 and eNOS / NOX4**					
51	**Chen ZW et al., 2018**	Human vascular ECs exposed to uraemic serum	Pterostilbene (stilbenoid NRF2 activator via KEAP1/NRF2/HO-1 pathway)	Pterostilbene restored eNOS function and expression alongside reduced ROS and increased HO-1 and SOD. NRF2-mediated ROS reduction allows eNOS to remain coupled and NO-producing.	eNOS/NO: demonstrates NRF2 activation restores eNOS coupling in oxidatively stressed ECs, relevant to preserving NO bioavailability and VSMC quiescence in arterialised vein grafts.
52	**Huang et al., 2021**	Endothelial cells under high glucose (type 2 diabetic model)	HDAC3 pharmacological inhibition; NOX4 siRNA knockdown	HDAC3 inhibition activated NRF2 and suppressed NOX4 transcription at source. NOX4 silencing reciprocally increased NRF2, HO-1, and NQO1. HDAC3 is elevated in diabetes, suppressing NRF2 basally.	NOX4/eNOS: NRF2-mediated NOX4 transcriptional repression is a sustainable ROS source-control strategy. HDAC3 inhibition is a specific therapeutic approach for diabetic CABG patients with suppressed baseline NRF2.
50	**Xue et al., 2017**	Skin endothelial cells; murine radiation-induced injury model	NRF2/GCH1/BH4 signalling axis activation and inhibition	NRF2 upregulates GCH1 (rate-limiting BH4 synthesis enzyme), increasing BH4. Preserved BH4 maintains eNOS coupling. NRF2/GCH1/BH4 axis ameliorated oxidative vascular injury.	eNOS/NO module: mechanistic basis for NRF2-mediated BH4 restoration, interrupting the peroxynitrite-driven eNOS uncoupling loop that perpetuates nitrosative injury in VGD.
**RSS / H2S and KEAP1**					
55	**Tocmo & Parkin, 2019**	Primary murine hepatocytes	S-1-propenylmercaptocysteine	KEAP1 cysteine persulfidation by organosulfur compound stabilised NRF2 and activated ARE-	RSS/KEAP1: persulfidation is a mechanistically distinct NRF2 activation route complementary to ROS-mediated oxidation. Foundation for H2S-

Ref	Study (First Author, Year)	Model System	Intervention / Pathway Examined	Key Mechanistic Finding	Relevance to VGD / IH Pathway
			(organosulfur compound; KEAP1 persulfidation)	driven transcription of HO-1, NQO1, and glutathione synthesis enzymes.	supplementation strategies to compensate for arterial shear-driven CSE downregulation.
**56**	**Cortese-Krott et al., 2016**	Human vascular endothelial cells	Nitrosopersulfide (SSNO-, bioactive H2S/NO reaction product)	SSNO- activated NRF2 nuclear accumulation and HO-1 mRNA expression more potently than H2S or NO alone. Positions the H2S/NO interaction product as a potent endogenous KEAP1/NRF2 activator.	RSS axis: the H2S/NO interaction product is a more potent NRF2 activator than either molecule alone — highlighting why preserving both eNOS-derived NO and CSE-derived H2S is essential for optimal vein graft protection.
**57**	**Ling et al., 2019**	Rat balloon vascular injury (restenosis) model	Exogenous H2S donor compound; NRF2/HIF-1a pathway analysis	H2S donor enhanced NRF2 nuclear accumulation, upregulated HO-1 and SOD, and suppressed neointimal hyperplasia via NRF2/HIF-1a signalling.	RSS translational relevance: exogenous H2S activates NRF2 and suppresses IH in a vascular injury model, supporting H2S-releasing compounds in vein graft preservation solutions.
**58**	**Kip et al., 2020**	Murine vein graft model	GYY4137 (H2S-releasing pro-drug); local periprocedural topical delivery	GYY4137 reduced intimal:medial area ratio by more than 50% and VSMC migration by approximately 33%. Most direct vein graft evidence for H2S-based VGD prevention.	Translational (vein graft model): provides the strongest pre-clinical evidence for periprocedural RSS supplementation as a VGD-prevention strategy, supporting incorporation into modified preservation solutions.
**NRF2 and Endothelial Protection**					
**60**	**Zeng et al., 2018**	HUVECs exposed to H2O2-induced oxidative stress	MALAT1 lncRNA overexpression and knockdown	MALAT1 reduced KEAP1 mRNA, increased NRF2, HO-1, NQO1, and GCLC, and reduced oxidative apoptosis. MALAT1 knockdown enhanced apoptosis and suppressed NRF2 signalling.	Endothelial protection: NRF2 activation via lncRNA-KEAP1 axis prevents EC apoptosis. Early EC death exposes thrombogenic basement membrane and initiates IH — NRF2-mediated protection addresses this at the earliest stage.
**61**	**Bontor & Gabryel, 2024**	Human endothelial cells; 4-HNE-induced oxidative stress model	Sulodexide (NRF2 activator via sestrin2/NRF2 pathway)	Sulodexide activated NRF2, reduced pro-apoptotic Bax and cleaved caspase-3, and suppressed ROS. Strong inverse correlation between ROS and EC viability (R2=0.78, p<0.001).	Endothelial protection: NRF2-dependent protection against 4-HNE — a VGD-relevant oxidant from lipid peroxidation in grafted vein tissue that also drives VSMC MAPK activation.
**NRF2 and Angiogenesis**					
**23**	**Florczyk et al., 2014**	NRF2 knockout and overexpression ECs; murine hind limb ischaemia in vivo	NRF2 genetic knockout and overexpression; HIF-1a/VEGF pathway analysis	NRF2 supported physiological angiogenesis and EC survival under ischaemia while suppressing pathological neovascularisation. Regulated HIF-1a and VEGF in a context-dependent bidirectional manner.	Angiogenesis: NRF2 promotes vasa vasorum restoration after harvest-induced disruption while restraining pathological neovascularisation that delivers inflammatory cells to the intima and drives IH.
**62**	**Sthijns et al., 2017**	HUVECs and placental arterioles under oxidative stress (H2O2)	Rutin quinone (NRF2 activator via Cys151 KEAP1 modification)	Rutin quinone enhanced NRF2 transcriptional activity and simultaneously blunted HIF-1a stabilisation. Improved vascular relaxation and reduced oxidative impairment.	Angiogenesis/therapeutic targets: a Cys151-specific KEAP1 modifier that simultaneously activates NRF2 and suppresses HIF-1a — relevant

Ref	Study (First Author, Year)	Model System	Intervention / Pathway Examined	Key Mechanistic Finding	Relevance to VGD / IH Pathway
					to the NRF2/angiogenesis balance in arterialised vein grafts and as a therapeutic candidate.
**NRF2 and EndMT**					
**69**	**Ryoo et al., 2014**	Renal tubular epithelial cells under TGFb1 stimulation	KEAP1-NRF2 pathway activation and inhibition; SMAD signalling analysis	KEAP1-NRF2 pathway inhibited TGFb1-stimulated epithelial-to-mesenchymal transition. NRF2 activation modulated SMAD signalling and suppressed mesenchymal marker expression.	EndMT: mechanistic precedent for NRF2/KEAP1 antagonism of TGFb/SMAD-driven mesenchymal transition. Supports the proposed NRF2-EndMT suppressive axis in vein grafts (not yet directly tested in LSV).
**70**	**Chen Y et al., 2017**	Pulmonary vascular ECs; pulmonary hypertension/vascular remodelling model	NRF2 activation via plant polyphenol (resveratrol)	NRF2 activation attenuated pulmonary vascular remodelling by inhibiting EndMT, preserving endothelial markers and suppressing mesenchymal marker upregulation via TGFb/SMAD pathway suppression.	EndMT: vascular endothelial evidence that NRF2 activation suppresses EndMT via TGFb/SMAD — the same pathway operative during arterialisation of vein grafts, strongest indirect vascular support for this therapeutic strategy.
**71**	**Chen F et al., 2024**	Multiple cell lines; biochemical KEAP1 isoform binding assays	KEAP1 stable knockdown; NRF2/SMAD2/3 competitive binding domain analysis	KEAP1 physically interacts with SMAD2/3 via motifs homologous to NRF2 binding domains, sequestering SMAD2/3 in the cytoplasm. KEAP1 knockdown suppressed TGFb-stimulated SMAD2/3 phosphorylation and fibrotic gene expression.	EndMT: identifies a novel direct KEAP1-SMAD2/3 physical interaction that positions KEAP1 as a dual gatekeeper of NRF2 stability and SMAD2/3-driven EndMT — reframing KEAP1 as a molecular integrator beyond its NRF2 regulator role.
**72**	**Yuan et al., 2024**	Intestinal fibrosis model (inflammatory bowel disease)	NRF2/HO-1 activation; NRF2 agonist treatment	NRF2 prevents fibroblast-to-myofibroblast transformation by inhibiting ROS-dependent TGFb/SMAD signalling. NRF2 agonists suppressed TGFb levels and fibrotic gene expression (fibronectin-1, collagen 1A1).	EndMT/fibrosis: NRF2 suppression of fibrotic transformation via ROS-TGFb/SMAD supports the proposal that NRF2 agonists could suppress EndMT-driven fibrotic gene expression in vein grafts.
**73**	**Liu Y et al., 2017**	Intestinal mucosal ECs; traumatic brain injury model	ERK/NRF2/HO-1 cascade activation and inhibition	NRF2/HO-1 activation via ERK preserved endothelial barrier integrity by enhancing tight junction protein expression. HO-1 products (CO, biliverdin) exerted additional anti-inflammatory and anti-apoptotic effects.	Endothelial barrier/EndMT: NRF2/HO-1 preservation of tight junction integrity directly relevant to preventing early EC barrier disruption in vein grafts, which precedes inflammatory infiltration and the IH cascade.
**Therapeutic Targets**					
**85**	**Heyninck et al., 2016**	HUVECs	Withaferin A (Cys151-targeting KEAP1 inhibitor)	Withaferin A induced robust HO-1 expression in ECs via Cys151-specific KEAP1 modification and NRF2 activation, without broad transcriptomic off-target effects.	Therapeutic targets: identifies withaferin A as a Cys151-selective post-translational KEAP1 inhibitor with endothelial evidence, supporting reversible targeted NRF2 stabilisation as the most promising peri-operative VGD-prevention strategy.
**Haemodynamic Forces and IH**					

Ref	Study (First Author, Year)	Model System	Intervention / Pathway Examined	Key Mechanistic Finding	Relevance to VGD / IH Pathway
67	**Yao et al., 2023**	Murine vein graft model under oscillatory shear stress	NADPH oxidase / AKT/BIRC5 signalling pathway analysis	Oscillatory shear promoted vein graft IH via NOX-mediated ROS generation, activating AKT/BIRC5 survival signalling in VSMCs and driving proliferation, migration, and apoptosis resistance.	NOX4/MAPK: directly demonstrates that disturbed flow at anastomoses drives VSMC IH via NOX-ROS — the same pathway NRF2-mediated NOX4 transcriptional repression would interrupt, explaining focal IH at anastomotic sites.
65	**Grudzinska et al., 2018**	Incompetent great saphenous vein segments from patients with chronic venous disease	Cytokine profiling in incompetent vs laminar-flow LSV from same patients	Elevated IL-1b, IFN-g, IL-2, IL-4, IL-8, IL-12, and MCP-1 in incompetent LSV vs laminar flow veins in the same patients. Flow-dependent inflammatory phenotype.	Inter-patient variability: pre-existing chronic venous disease creates chronically inflamed LSV before CABG, suggesting maladaptive p62-mediated NRF2 activation may already be present in some patients at surgery.
66	**Zamboni et al., 2016**	Chronic venous disease patients before and after surgical flow correction (CHIVA procedure)	Surgical restoration of laminar flow; cytokine profiling before and after	Oscillatory flow suppression significantly reduced vein wall cytokine burden after surgical correction. The inflammatory phenotype was flow-dependent and at least partially reversible.	Haemodynamic forces: demonstrates the inflammatory state of LSV is flow-determined and reversible, supporting the concept that laminar flow regions in vein grafts may sustain protective NRF2 signalling via ARE-mediated anti-inflammatory gene expression.
**Inter-Patient Variability (Diabetes / Epigenetics / Sex)**					
77	**Chen X et al., 2020**	Vascular ECs under high glucose conditions	SET8 (histone H4K20 monomethyltransferase) expression modulation; KEAP1 promoter regulation	High glucose inhibited KEAP1/NRF2/ARE signalling via downregulation of SET8. SET8 maintains KEAP1 expression through H4K20 monomethylation. Loss of SET8 reduces NRF2 responsiveness.	Diabetes/epigenetics: identifies an epigenetic mechanism suppressing NRF2 in diabetic ECs via SET8/KEAP1 — relevant to the reduced protective NRF2 threshold in diabetic CABG patients. SET8 is hormonally regulated, linking sex and metabolic risk factors.
78	**Ishii & Warabi, 2019**	Endothelial cells; membrane-associated oestrogen receptor signalling	Oestrogen receptor activation; NOX1, neutral sphingomyelinase 2, and EGFR pathway analysis	Oestrogen activates NRF2 via rapid non-genomic membrane-associated oestrogen receptor mechanisms involving NOX1, neutral sphingomyelinase 2, and EGFR. Loss of oestrogen removes this NRF2 activation pathway.	Sex/inter-patient variability: non-genomic oestrogen-NRF2 activation pathway is lost with menopause, providing mechanistic basis for reduced NRF2 protective responses in post-menopausal female CABG patients contributing to worse vein graft outcomes in women.

Abbreviations: 4-HNE, 4-hydroxynonenal; ARE, antioxidant response element; BH4, tetrahydrobiopterin; CABG, coronary artery bypass grafting; CBP/p300, CREB-binding protein; CSE, cystathionine gamma-lyase; EC, endothelial cell; EndMT, endothelial-to-mesenchymal transition; GCLC/M, glutamate-cysteine ligase catalytic/modifier subunit; GCH1, GTP cyclohydrolase 1; HDAC3, histone deacetylase 3; HIF-1a, hypoxia-inducible factor 1-alpha; HO-1, haem oxygenase-1; HUEC, human umbilical vein endothelial cell; IH, intimal hyperplasia; KEAP1, kelch-like ECH-associated protein 1; lncRNA, long non-coding RNA; LSV, long saphenous vein; MAPK, mitogen-activated protein kinase; NFkB, nuclear factor kappa-B; NQO1, NAD(P)H quinone dehydrogenase 1; NRF2, nuclear factor erythroid 2-related factor 2; NOX4, NADPH oxidase 4; RSS, reactive sulfur species; SLC7A11, solute carrier family 7 member 11; SMAD, small mothers against decapentaplegic; SOD, superoxide dismutase; SSNO-, nitrosopersulfide; TGFb, transforming growth factor beta; VGD, vein graft disease; VSMC, vascular smooth muscle cell.
